# Fabricating niosomal-PEG nanoparticles co-loaded with metformin and silibinin for effective treatment of human lung cancer cells

**DOI:** 10.3389/fonc.2023.1193708

**Published:** 2023-08-17

**Authors:** Elnaz Salmani-Javan, Davoud Jafari-Gharabaghlou, Esat Bonabi, Nosratollah Zarghami

**Affiliations:** ^1^ Department of Clinical Biochemistry and Laboratory Medicine, Faculty of Medicine, Tabriz University of Medical Sciences, Tabriz, Iran; ^2^ Department of Medical Microbiology, Faculty of Medicine, Istanbul Aydin University, Istanbul, Türkiye; ^3^ Department of Medical Biochemistry, Faculty of Medicine, Istanbul Aydin University, Istanbul, Türkiye

**Keywords:** niosomal nanoparticles, metformin, silibinin, lung cancer, hTERT

## Abstract

**Background:**

Despite current therapies, lung cancer remains a global issue and requires the creation of novel treatment methods. Recent research has shown that biguanides such as metformin (MET) and silibinin (SIL) have a potential anticancer effect. As a consequence, the effectiveness of MET and SIL in combination against lung cancer cells was investigated in this study to develop an effective and novel treatment method.

**Methods:**

Niosomal nanoparticles were synthesized via the thin-film hydration method, and field emission scanning electron microscopy (FE-SEM), Fourier transform infrared (FTIR), atomic force microscopy (AFM), and dynamic light scattering (DLS) techniques were used to evaluate their physico-chemical characteristics. The cytotoxic effects of free and drug-loaded nanoparticles (NPs), as well as their combination, on A549 cells were assessed using the MTT assay. An apoptosis test was used while under the influence of medication to identify the molecular mechanisms behind programmed cell death. With the use of a cell cycle test, it was determined whether pharmaceutical effects caused the cell cycle to stop progressing. Additionally, the qRT-PCR technique was used to evaluate the levels of hTERT, BAX, and BCL-2 gene expression after 48-h medication treatment.

**Results:**

In the cytotoxicity assay, the growth of A549 lung cancer cells was inhibited by both MET and SIL. Compared to the individual therapies, the combination of MET and SIL dramatically and synergistically decreased the IC50 values of MET and SIL in lung cancer cells. Furthermore, the combination of MET and SIL produced lower IC50 values and a better anti-proliferative effect on A549 lung cancer cells. Real-time PCR results showed that the expression levels of hTERT and BCL-2 were significantly reduced in lung cancer cell lines treated with MET and SIL compared to single treatments (p< 0.001).

**Conclusion:**

It is anticipated that the use of nano-niosomal-formed MET and SIL would improve lung cancer treatment outcomes and improve the therapeutic efficiency of lung cancer cells.

## 1 Introduction

In the last century, the most prevalent malignancy is lung cancer, which causes the most mortality from cancers (1). Statistical analysis revealed that lung cancer caused 19% of cancer deaths (2). According to the biology, prognosis, and therapy responsiveness of lung cancer, the World Health Organization (WHO) has categorized it into two groups: small cell lung cancer (SCLC) and non-small cell lung cancer (NSCLC) (3). Almost 85% of types of lung cancer are of the last group (4). Lung cancer has a high prevalence in both men and women (5), but in most countries, men have higher mortality from lung cancer causes (6). Risk factors such as smoking genetics, diet, alcohol, ionizing radiation, air pollution (2), and behavioral and environmental factors (1) are causes of lung cancer (1). However, smoking is considered the most significant risk factor (2). Traditional chemotherapy is limited because of its adverse effects and high cytotoxicity; as a result, the development of another treatment could limit the side effects of the treatment (7). Recent studies recommend a combination of two or more therapeutic compounds to decrease adverse side effects, elevate the modality of life, decrease drug resistance and population of cancer stem cells, reduce metastasis, and improve the lifetime of patients (7, 8). A recent study has shown that the treatment of human colorectal cancer cell lines with metformin (MET) and silibinin (SIL) inhibits cell survival and induces apoptosis (7). SIL is a polyphenolic flavonolignan and natural antioxidant found in *Silybum marianum*. It has anticancer effects in certain cancer forms, like breast, prostate, kidney, skin, pancreas, bladder, lung, and colon cancers. It also has hepato-protective, immune-stimulatory, cell cycle arrest, and antioxidant effects (9, 10) and causes apoptosis activation in various cancer cells (11). SIL exerts anticancer influence through several signaling pathways and molecules including VEGF, VEGF receptors, NF-kB, IGF-IGFBP3, b-catenin, PI3K/Akt, STAT, AMPK, and MAPK [9]. MET is another natural antioxidant that is being applied to manage type 2 diabetes (12, 13). The anticancer effect of MET is mediated by AMPK (13). Also, MET modulates the AMPK/mTOR/p70S6K pathway; inhibits protein synthesis (8, 14), cell mitosis, and proliferation (15, 16); and suppresses metastasis (17). Current studies have shown that in a dose-dependent manner, MET and SIL can suppress the expression of the human telomerase reverse transcriptase (hTERT) gene (18, 19). hTERT gene expression regulates telomerase function (20), produces the telomerase catalytic subunit (21), and keeps the length of the telomere (20, 22). It has been found that telomerase is activated in almost 85%–90% of the most prevalent tumor including pancreatic, prostate, liver, breast, colon, and lung cancers (3, 23). Accordingly, telomerase has been considered a potential treatment target for treating different types of cancers (8, 24, 25). The latest studies have identified BAX and BCL-2 as other genes that influence the survival of cancer cells (26). Bcl-2 is considered a vital regulator and inhibitor of apoptosis (27, 28). It has been revealed that during apoptosis, the expression of BAX is upregulated, the expression of BCL-2 is downregulated, and the ratio of BAX/BCL-2 seems to be important for inducing apoptosis (28, 29). However, a high level of BCL-2 than BAX causes more susceptibility to apoptosis (28, 29). Injected chemotherapy drugs have several insufficiencies including severe side effects, low bioactivity, drug resistance, quick degradation, and clearance from the body. These limitations could be improved using nanoparticles (NPs) as their drug delivery system (30, 31). Different nanoparticles are used in drug delivery, but among them, niosome has been identified as one of the most suitable carriers (32). Niosome is a degradable, biocompatible, non-immunogenic, and harmless carrier composed of non-ionic surfactants and lipid compounds, which could transfer hydrophilic and hydrophobic drugs. Unlike conventional drug delivery forms, the release rate of niosome could be controlled by modifying its composition or surface (33, 34). Polyethylene glycol (PEG) has been approved as a safe surface modifier due to its being biocompatible, hydrophilic, flexible, and non-toxic effects (35). To increase circulatory stability and half-life, niosome could be covered with PEG to form PEGylated niosome. Regarding the anticancer effect of MET and SIL, in this research, we investigate the synergistic effect of co-delivery MET and SIL loaded in PEGylated niosome to enhance their bioavailability and evaluate their effects on the expression of hTERT, BCL-2, and BAX genes in NSCLC type lung cancer cells.

## 2 Material and methods

### 2.1 Cell line and chemicals

A549 lung cancer cell line and human embryonic kidney 293 (HEK 293) were acquired from the Institut Pasteur Cell Bank, Iran (Cat. No C203), and maintained according to recommendations. Metformin (1,1-dimethyl biguanide hydrochloride), silibinin, dimethyl sulfoxide, 3-(4,5-dimethylthiazol-2-yl)-2,5-diphenyltetrazolium bromide (MTT), PEG (Mw = 4,000), dimethyl sulfoxide (DMSO), polyvinyl alcohol (PVA) and cholesterol, non-ionic surfactants (Span-60), methanol, and chloroform were purchased from Sigma Aldrich (St. Louis, MO, USA). Fetal bovine serum (FBS), penicillin–streptomycin, and RPMI-1640 were taken from Gibco BRL (Gaithersburg, MD, USA). The first strand of complementary DNA (cDNA) synthesized with the kit purchased from Fermentas (Vilnius, Lithuania) and Syber Green PCR Master Mix kit was provided from Roche (Mannheim, Germany).

### 2.2 Metformin- and silibinin-loaded PEGylated-niosome synthesis

First, PEG (3 mg), cholesterol (6 mg), and Span-60 (36 mg) were dissolved in 10 ml of an ethanol/chloroform mixture. The solvent was evaporated using a rotary evaporator at 120 rpm and 60°C under reduced pressure. After the evaporation of the organic solvent, a thin film was formed at the bottom of the round-bottom flask. Then, the formed layer was hydrated with phosphate-buffered saline (PBS) containing MET (1.6 mg) and evaporated by a rotary evaporator at 90 rpm and 60°C. The obtained solvent was collected and sonicated using a sonicator for 30 min to get a uniform, smaller, and more homogenized niosome. To provide SIL-loaded PEGylated niosome, SIL (4.8 mg) was dissolved in a mixture of chloroform–ethanol with niosome. The samples were prepared and kept at 4°C for later analyses.

### 2.3 Size and zeta potential assessments

Dynamic light scattering (DLS) technique (Nano ZS, Malvern Instruments Ltd., Malvern, UK) was applied to evaluate the average size and surface charge of the prepared niosome. In summary, the niosome samples were properly diluted (1:10) with deionized water, and the sample size and zeta potential were measured according to the DLS method using Zetasizer Nano ZS (Malvern Instruments Ltd., Malvern, UK) with a helium–neon laser at 630 nm in room temperature.

### 2.4 FE-SEM

A field emission scanning electron microscopy (FE-SEM) model AIS 2100 (Seron Technologies, Gyeonggi-do, Korea) was used to examine the surface morphology and microstructure of synthesized PEGylated niosome. A small number of samples were used for this, which were rinsed twice with pure water, freeze-dried, and scanned using an electron microscope.

### 2.5 Determination of entrapment efficiency

Spectrophotometry was applied to evaluate the entrapment efficacy (EE). Initially, various doses of MET and SIL in methanol were used to adjust the calibration curve. After that, Amicon MPS (Millipore, Burlington, MA, USA) was used to load a niosome solution and centrifuged (Hettich, Tuttlingen, Germany). The entrapped niosome remained at the top of the tube membrane, while the supernatant was detached from the bottom filter cup. The concentration of free MET and free SIL in the supernatant was recorded using absorbance measurements at 234 and 287 nm, respectively. The percentage of entrapment efficiency was calculated by the following formula:


$$\text{EE\%}=\frac{\text{Total amount of MET}/\text{SIL}-\text{free MET}/\text{SIL}}{\text{Total amount of MET}/\text{SIL}}\times 100.$$

### 2.6 FTIR analysis

In order to distinguish between the loaded and unloaded drug in the niosome, Fourier transform infrared spectroscopy (FTIR) was used. After lyophilized into a dry powder, the samples were combined with potassium bromide. The specimens were then put into a hydraulic press to create pellets. The samples were scanned in the wavelength range of 400–4,000 cm^−1^.

### 2.7 Cell culture

A549 and HEK 293 cell lines were purchased from the Institut Pasteur cell culture collection (Tehran, Iran). The cells were grown in RPMI-1640 medium complemented with FBS (10%), 0.05 mg/ml of penicillin G, and 0.08 mg/ml of streptomycin. Cells were placed in a sterile flask and incubated at 37°C in a humidified atmosphere containing 5% CO_2_.

### 2.8 Investigation of *in vitro* drug release

With the use of a dialysis bag (MW = 12 kDa), the *in vitro* release of MET and SIL from the niosome was assessed. A specified volume of MET-NP and SIL-NP was dispersed in PBS with pH values of 4.4 and 7.4, respectively. The release of both drugs was estimated in PBS while continuously agitating the MET and SIL niosome samples in the dialysis tubes. After that, the sample was periodically removed from the incubation medium and quickly replaced with an equivalent amount of fresh PBS. A UV–Vis spectrometer was applied to measure the release of MET and SIL at 234 and 287 nm, respectively.

### 2.9 Cytotoxic test

With the use of MTT assay, the cytotoxic properties of drugs were evaluated in their free, loaded, and combination forms. In summary, a 96-well plate was seeded with 2 × 10^4^ A549 lung cancer cells per well and incubated for 24 h at 37°C in a humidified atmosphere containing 5% CO_2_ to encourage cell adhesion. Furthermore, we applied human embryonic kidney 293 (HEK 293) as a normal cell line and evaluated the cytotoxicity of niosomal metformin and silibinin on this cell line. Then, the cells were exposed to various doses of the free and loaded forms of MET and SIL and their combinations listed in **Table 1** for 48 h. Also, a group of cells was treated with a blank niosome as a control. Following the incubation period, 200 μl of the phosphate buffer solution containing 0.5 mg/ml of MTT was added to the cell culture wells, and the plates were then covered with an aluminum foil and incubated for 4 h at 37°C. After the addition of 200 μl of pure DMSO and 25 μl of Sorensen’s glycine buffer, the entire contents of the wells were then removed. The resulting formazan crystals were then extracted after 20 min of incubation. Finally, using an ELISA microplate reader (BioTek Power Wave XS) with a reference wavelength of 630 nm, the absorbance of formazan at 570 nm was measured, and the number of living cells was evaluated. There were three separate runs of each experiment.

**Table 1:** Amounts of each compound used for cytotoxicity test.

SIL (μM)	MET	SIL (μM)/MET (mM)	SIL-NPs (μM)		MET-NPs (μM)	SIL/MET-NPs (μM)	
5	5	5/5	5		25	5/25
10	10	10/10	10		50	10/50
15	15	15/15	15		100	15/100
30	30	30/30	30		150	30/150
40	40	40/40	40		200	40/200

MET, metformin; SIL, silibinin; NPs, nanoparticles.

### 2.10 RNA extraction, cDNA synthesis, and real-time PCR

A real-time PCR test was applied to assess the transcription mRNA of hTERT, BAX, and BCL-2 genes. First, 2 × 10^6^ A549 cells were seeded in six-well plates and incubated at 37°C with 5% CO_2_. After 24 h, cells were exposed separately to free-MET (F-MET), free-SIL (F-SIL), nano-MET (N-MET), nano-SIL (N-SIL), and a combination of F-MET-SIL and N-MET-SIL at their IC50 concentration. After 48-h treatment of free and drug-loaded NPs, total mRNA was extracted by TRIzol (Sigma, Roedermark, Germany) reagent based on the instrument. Then, using measurements of the OD260/280 ratio, the quantity and quality of the total extracted mRNA were determined. Then, employing a first-strand cDNA synthesis kit (Fermentas, Vilnius, Lithuania), cDNA was synthesized from mRNA extracted from each sample in accordance with the manufacturer’s instruction. According to the manufacturer’s recommendations, hTERT, BAX, and BCL-2 gene expression levels were then assessed using the qPCR method employing the Hot Taq EvaGreen qPCR. The primer-blast on the National Center for Biotechnology Information (NCBI) website was used to blast the exact primers that were utilized for real-time PCR (**Table 2**). The program for real-time PCR was as follows; the first step was denaturation at 95°C for 10 min, followed by cycles of denaturation at 95°C for 15 s (1 cycle), annealing step at 60°C for 30 s (40 cycles), extension step at 72°C for 30 s (40 cycles), and melting step at 65°C–95°C (1 cycle). In the end, the housekeeping gene (GAPDH) was used to normalize the relative expression levels of hTERT, BAX, and BCL-2.

**Table 2:** Primer sequences used for real-time PCR amplification.

Genes	Forward (5′→3′)	Reverse (5′→3′)
hTERT	TTCCTGCACTGGCTGATGAGT	AGAAAGACCTGAGCAGCTCGAC
BAX	ACGTGGGCATTTTTCTTACTTT	TATTACCCCCTCAAGACCACT
BCL-2	AAACTTGACAGAGGATCATGC	ATCTTTATTTCATGAGGCACGTT
GAPDH	TCTGACTTCAACAGCGACACC	AAATGAGCTTGACAAAGTGGT

### 2.11 Apoptosis analysis

Apoptosis induction was investigated by F-MET, F-SIL, N-MET, N-SIL, and their combination on the A549 cell line using a flow cytometer and Annexin V staining method. In summary, six-well plates containing 5 × 10^5^ A549 cells were used to treat drugs in free form and nano-form and their combination. After 48 h of drug treatment, cells were harvested using trypsin (Sigma, Germany) and washed three times with PBS solution. Then, a certain amount of fixed solution was used to fix the cells. Annexin, V-FITC, and propidium iodide (PI) were then used to stain the cells, and they were incubated for 20 min. A flow cytometer was used to measure the cell mortality rate.

### 2.12 Cell cycle analysis

A total of 5 × 10^5^ A549 cells were seeded per well of six-well plates and incubated for 24 h. Then, cells were treated with F-MET, F-SIL, N-MET, N-SIL, and their combinations. Following 48 h of drug exposure, the cells were collected and washed three times with PBS before being fixed with 70% ethanol and stored at −20°C for 48 h. The cells were taken, re-suspended in PBS, and stained with a solution that contains RNase A and PI; then, the cells were incubated for 30 min at 37°C. The fluorescence was read using the flow cytometer.

### 2.13 Statistical analysis

Statistical significance and multi-group comparison of data were performed using a two-way analysis of variance (ANOVA) followed by Tukey’s *post-hoc* test using GraphPad Prism (version 8). Results with p ≤ 0.05 were regarded as statistically significant and were represented as the mean ± standard deviation (SD). All experimental trials were conducted three times and reported as mean ± SD.

## 3 Results and discussion

### 3.1 Size, morphology, and drug loading

**Figure 1:**
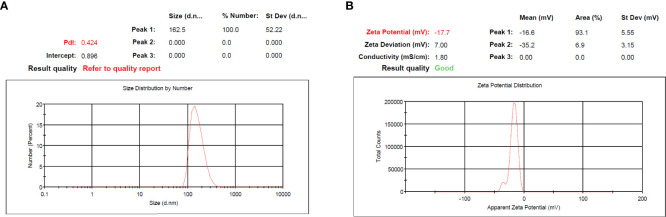
**(A)** PDI and **(B)** zeta potential of PEGylated-N-MET-SIL. PDI, polydispersity index; MET, metformin; SIL, silibinin.

According to guidelines, which is a key method for creating therapeutic NPs, MET and SIL were loaded in niosome NPs (36, 37). In cancer treatment, the combination of chemotherapy agents is the first line of cancer treatment. However, due to low stability and unwanted side effects (such as damage to intact cells, hair loss, loss of appetite, diarrhea, and drug resistance), combination chemotherapy is limited. NPs open up new possibilities for cancer treatment (38). The improvement of the bioavailability of drugs that are poorly water-soluble, the co-delivery of two or more drugs, the combination of therapeutic agents, targeted drug delivery, and particularly the accumulation of drugs at the site of the tumor (39), and controlling the release of drugs in target site are all possible with NPs (40). NPs are nano-sized, and for this reason, they can deeply penetrate objective tissues and be taken up by target cells (38). Niosome is a novel NP in the drug delivery system with considerable possibilities in numerous drug delivery systems (41, 42). Bilayer spherical vesicles called niosomes are formed of cholesterol and non-ionic surfactants in an aqueous medium. Cholesterol increases the stability and decreases vesicle permeability, increasing the effectiveness of the drug’s encapsulation (41). Because the drug carried in the niosome exclusively targets the intended cell and not non-target cells, the drug dose and toxicity can be minimized. By delaying the removal of medication molecules from circulation, niosomes enhance the therapeutic potential of drugs (42). Applying PEGylation in NPs is one of the most effective and extensively researched methods for enhancing the effectiveness of medications. The use of PEG in nano-formulations gives the nanoparticles hidden qualities, decreases the probability of removal by the reticuloendothelial system, and enhances the bioavailability and half-life of the medication utilized in the formulation (23, 43). PEGylation of the niosome increases their bioavailability. PEGylated niosomes slowly release drugs and can be hidden from the immune system; thus, they can circulate longer in the bloodstream. Additionally, it has been hypothesized that PEG’s high hydrophilicity causes niosomes to attach a coating of water to their surface, which hinders macrophages from recognizing and removing them. This change ultimately gives the niosomes more time *in vivo* to find their target sites before being digested by macrophages (44). To assess particle size and NP dispersion, the DLS method was used. As mentioned in **Table 3**, PEGylated niosome represented an average size of 124 ± 7.8 nm with a uniform size distribution, a polydispersity index (PDI) of 0.561, and zeta potential of −7.64 ± 4.2 mV. The average sizes of PEGylated-N-MET and PEGylated-N-SIL were 139 ± 4.1 and 147 ± 7.2 nm, respectively, with PDI of 0.912 and 0.596, respectively, and zeta potential of −24.37 ± 2.9 and −9.86 ± 3.7 mV, respectively. The size of N-MET and N-SIL was bigger than that of PEGylated niosome, which inferred that drugs were successfully loaded in NPs. PEGylated-N-MET-SIL displayed the largest size with 162.5 ± 1.8 nm (**Figure 1A**). The PDI of PEGylated-N-MET-SIL in comparison with N-MET and N-SIL due to the co-loading of MET and SIL was low, which led to the formation of the well-knit structures of the NPs. For the synthesized NPs, an average surface charge between −7.64 and −17.7 was found (**Figure 1B**). Smaller mean diameter NPs essentially migrate more quickly than bigger NPs in a known applied electric field. As a result, they have higher zeta potential values, which increases the stability of NPs in colloidal dispersion (45, 46).

**Table 3:** Characterization of PEGylated-niosome and drug-loaded NPs using DLS.

Groups	Size (nm)	Polydispersity index	Zeta potential (mV)
**PEGylated niosome**	124 ± 7.8	0.561	−7.64 ± 4.2
**MET-PEGylated niosome**	139 ± 4.1	0.912	−24.37 ± 2.9
**SIL-PEGylated niosome**	147 ± 7.2	0.596	−9.86 ± 3.7
**MET-SIL-PEGylated Niosome**	162.5 ± 1.8	0.424	−17.7 ± 7

NPs, nanoparticles; DLS, dynamic light scattering; MET, metformin; SIL, silibinin.

**Figure 2:**
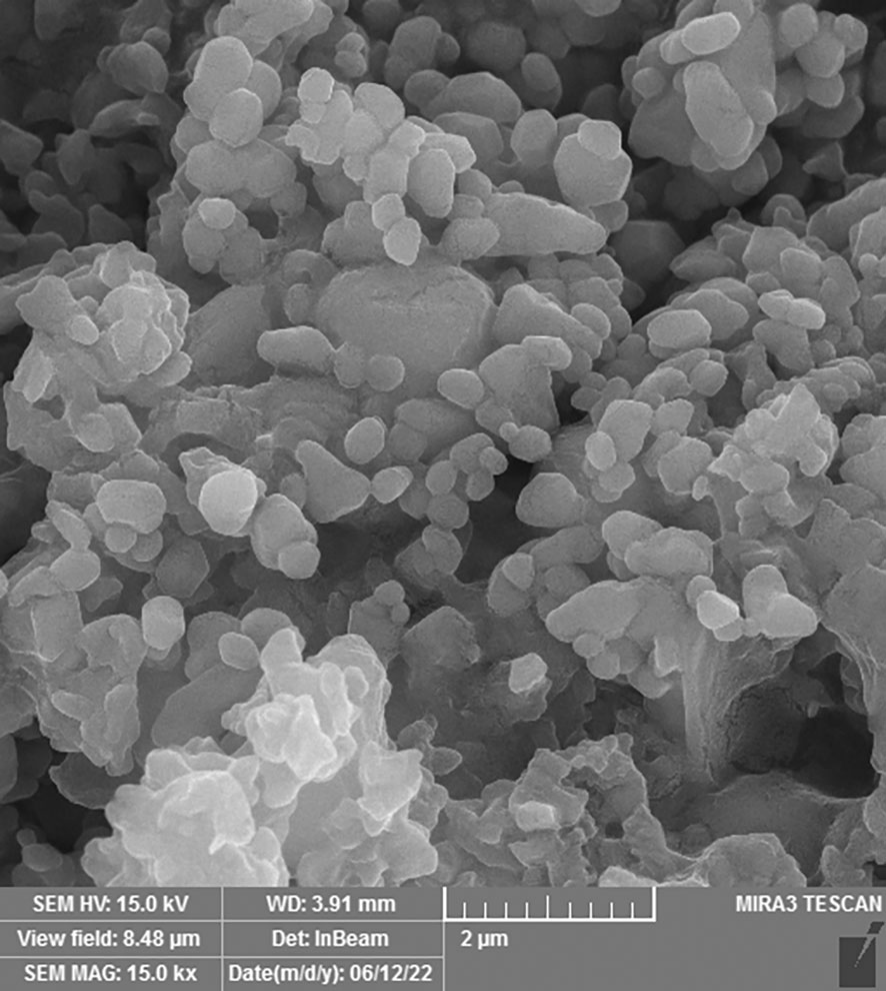
Field emission scanning electron microscopy (FE-SEM) analysis of PEGylated-N-MET-N-SIL displaying the core-shell structure of NPs. NPs, nanoparticles; MET, metformin; SIL, silibinin.

The SEM technique was used to examine the morphology of drug-loaded PEGylated-niosome NPs. The images of N-MET and N-SIL revealed an average diameter of approximately 145 nm and confirmed the drug’s spherical form and identical dispersion, which is shown in **Figure 2**.

**Figure 3:**
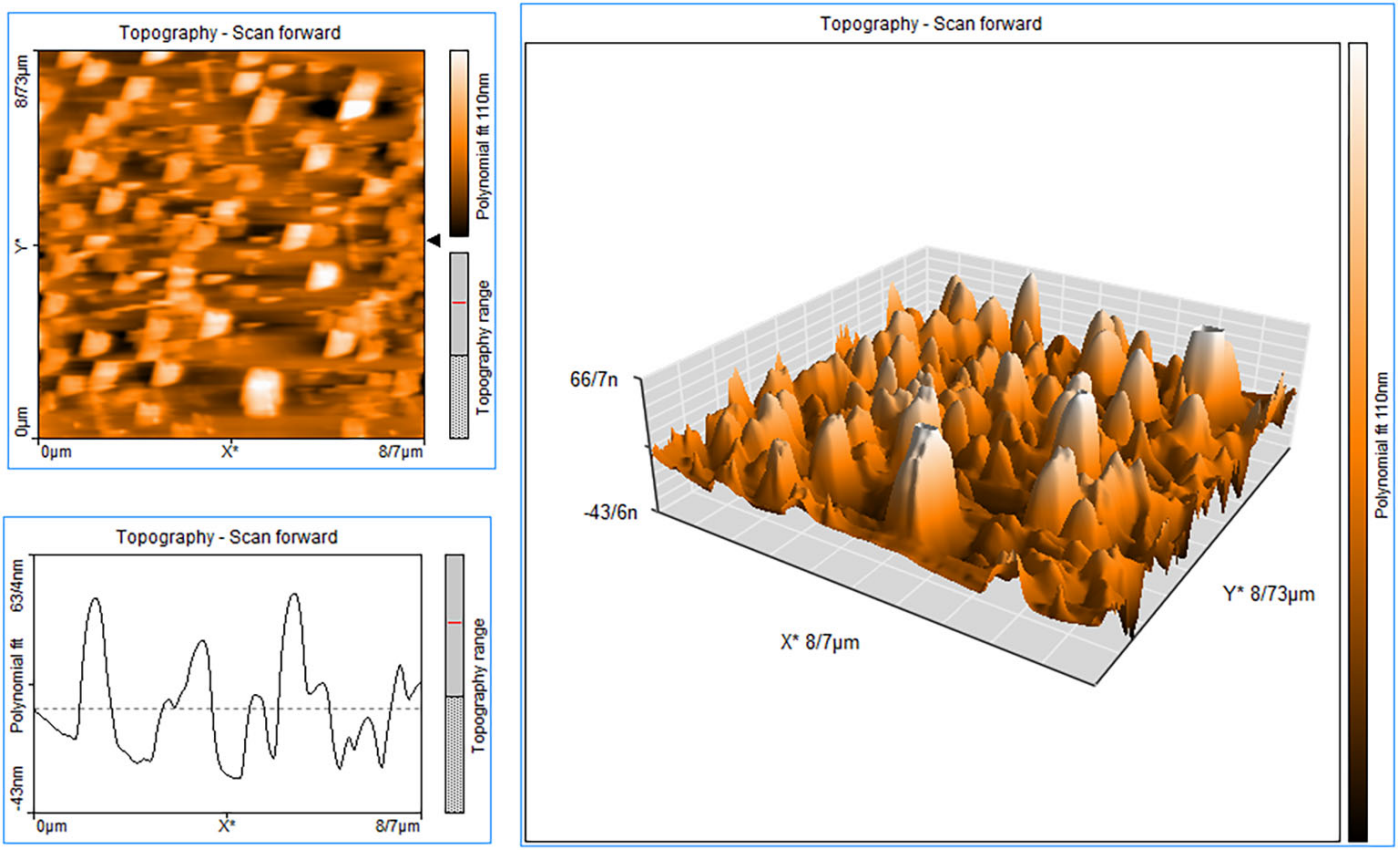
AFM image of N-MET-SIL indicated surface morphology of drug-loaded NPs. AFM, atomic force microscopy; NPs, nanoparticles; MET, metformin; SIL, silibinin.

According to the atomic force microscopy (AFM) analysis findings (**Figure 3**), which are in agreement with the SEM image and estimated size, particles with a height of approximately 66.7 nm from the surface are thought to be made of N-MET-SIL. Due to the use of a dehydrated sample of N-MET-SIL during the photography process, the size indicated by AFM was smaller than the results given by the DLS method.

**Figure 4:**
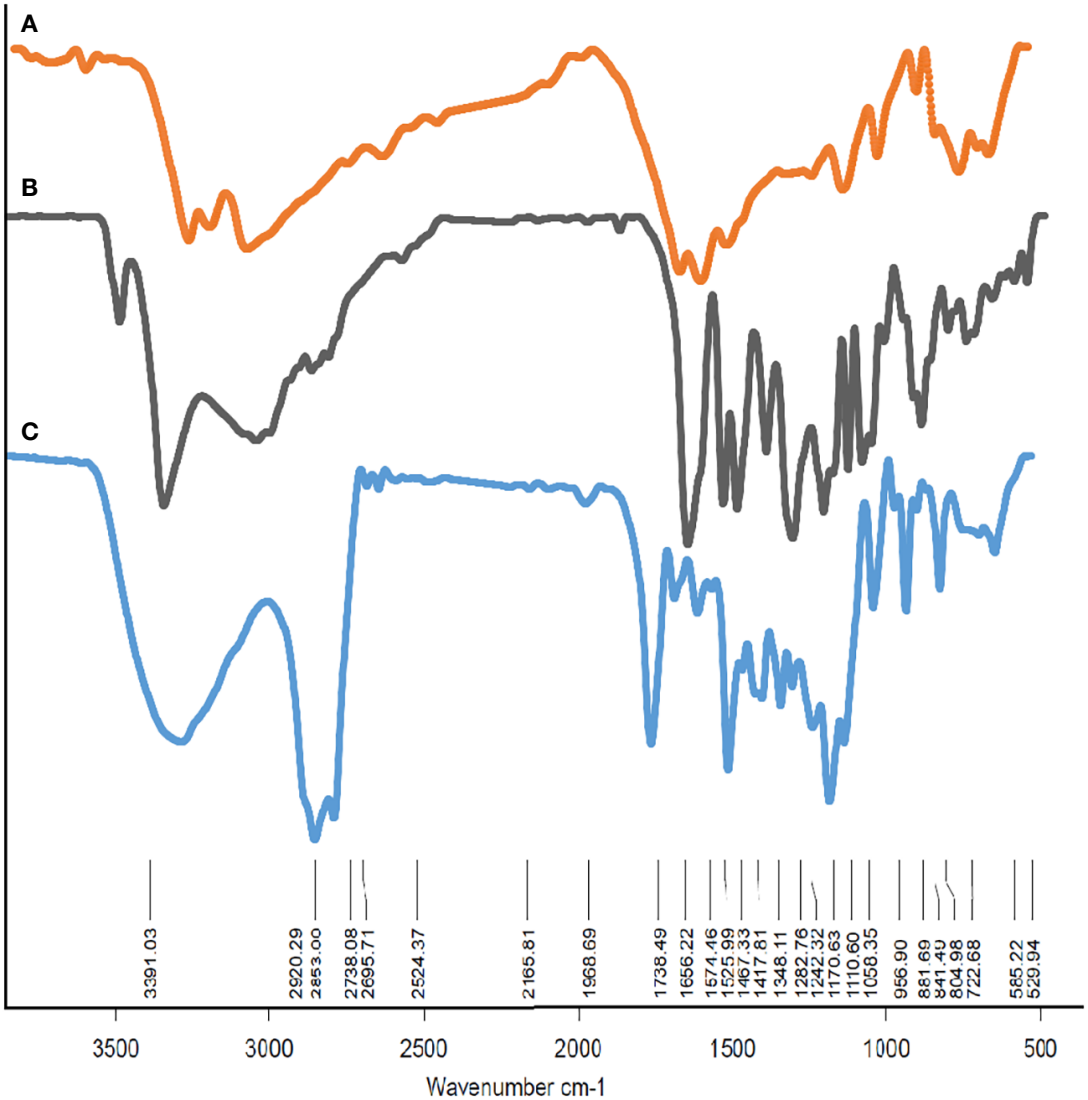
The FTIR spectrum of **(A)** F-MET, **(B)** F-SIL, and **(C)** PEGylated niosomal loaded MET-SIL. FTIR, Fourier transform infrared; MET, metformin; SIL, silibinin.

The drug encapsulation efficiency range was approximately 88.6%. To demonstrate the structure of F-MET and F-SIL and N-MET-SIL, FTIR spectroscopy was used (**Figure 4**). The FTIR spectrum of F-MET demonstrated two broad peaks at 3,300–3,600 cm^−1^ assigned to O–H and N–H bands, respectively, of polymer and drug. The peak at 2,362 cm^−1^ was related to MET C═N of imines stretching vibration. The peak at 2,362 cm^−1^ was related to MET C═N of imines stretching vibration. An intense peak at 1,759 cm^−1^ was the characteristic esoteric (O–C═O) bands of PEGylated niosome section. The peaks at 1,508–1,669 cm^−1^ are characteristic bands of NH primary bending, NH secondary bending, and C–N stretching vibrations of MET. Other characteristic peaks of –MET, which indicated C–C, C–O, and C–O–C, appeared at 1,059–1,187 cm^−1^. The FTIR spectrum of F-SIL showed a peak at 2,885 cm^−1^ attributed to C–H stretch of CH, and 3,010 and 2,955 cm^−1^ are due to C–H stretch of CH. A sharp peak at 1,630 cm^−1^ is attributed to C–O stretch. Absorption at 1,186–1,089.6 cm^−1^ is assigned to C–O stretch. The results display that MET and SIL have been successfully combined with PEGylated-niosome NPs. Our data were in agreement with the findings of Soumaye Amirsaadat et al. (47).

### 3.2 Drug release analysis

**Figure 5:**
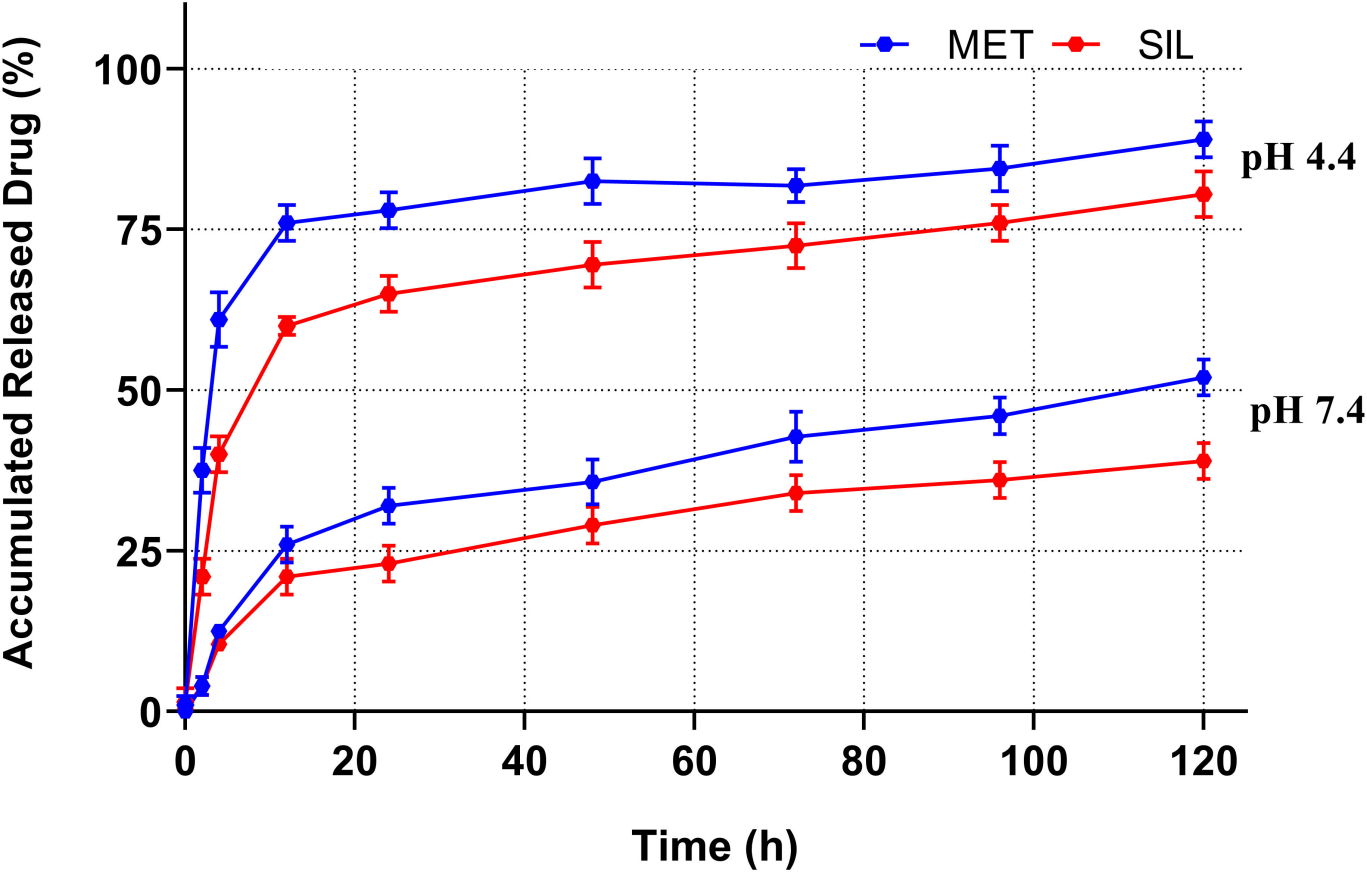
Drug release patterns of MET and SIL released from PEGylated-N-MET-SIL in PBS solution at pH 7.4 and 4.4. PBS, phosphate-buffered saline; MET, metformin; SIL, silibinin.

*In vitro*, drug release analyses were performed by using the dialysis method at pH of 7.4 and 4.4 (48). The cumulative proportion of MET and SIL released from NPs during various time periods is displayed in **Figure 5**.

Release of MET-NP and SIL-NP occurred in two phases. Initially, a controlled release was carried out for 6 h at a burst drug release rate, allowing for the release of 25% of the entrapped MET and SIL. After that, a continuous phase was carried out for 120 h at a reduced and slow release rate, and the maximum release of MET and SIL from PEGylated niosome reached 50% and 40% of the total entrapped drug, respectively, which indicated that persistency of MET and SIL in niosome at pH 7.4 is acceptable. This two-phase release pattern is in accordance with previous observations and seems to be a characteristic of bilayer vesicles in general (49).

### 3.3 *In vitro* cytotoxicity and synergistic analysis

The MTT test is one of the best techniques to evaluate the cytotoxic effects of drugs on cell proliferation (48). After 48-h drug treatment, an MTT test was performed to estimate the synergistic inhibitory and cytotoxic properties of MET and SIL in free form and nano-form and their combination. According to recent research, combining chemotherapeutic medicines rather than using them separately may be more effective at preventing the growth of tumors and lowering drug toxicity. With this strategy, drug resistance and ineffective drug use can be reduced, and therapeutic medication effectiveness can be increased (50). Our results demonstrated that MET and SIL could inhibit the growth and development of A549 cells, but the combination of MET/SIL niosome and F-MET/SIL had greater synergistic effects in comparison to the individual agents. These combinations were also able to conquer the toxicity and other side effects of a single agent and significantly suppress the development of the A549 cell line in a time- and dose-dependent manner (51). The blank niosome does not show any significant effect on the cells even in high doses. According to the result of the MTT assay, the blank niosome treatment group was excluded from the rest of the tests.

**Figure 6:**
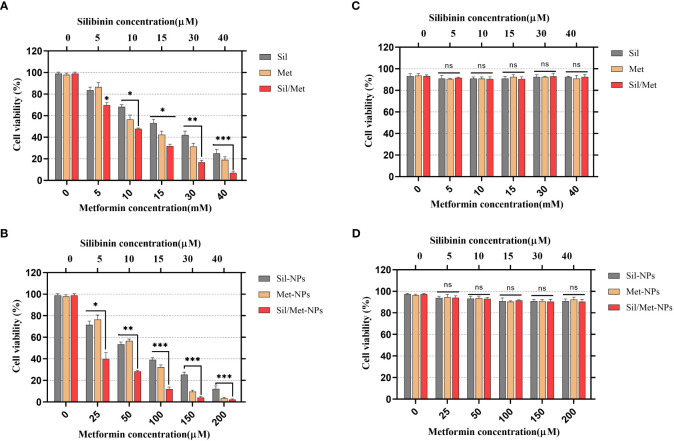
Effects of MET, SIL, and their nano-form on A549 lung cancer cell viability. **(A)** A549 cell line was treated with F-MET and F-SIL and their combination. **(B)** A549 cell line was treated with PEGylated-N-MET, PEGylated-N-SIL, and their combination. **(C)** HEk-293 cell line was treated with F-MET and F-SIL and their combination. **(D)** HEk-293 cell line treatment with PEGylated-N-MET, PEGylated-N-SIL, and their combination. Cell viability was measured using MTT assay after 48-h treatment. Data represented are from three independent experiments (***p< 0.001, **p< 0.01, and *p< 0.05). MET, metformin; SIL, silibinin; and ns, not significant.

As shown in **Figure 6**, due to delayed release and improved drug absorption from the niosome, the cytotoxic effects of medicines in N-MET and N-SIL are greater than F-MET and F-SIL in the A549 cell line. Moreover, the treatment of HEK 293 cells with the applied agents demonstrated no significant cytotoxicity. It suggests that by improving MET’s and SIL’s solubility and bioavailability, this form of drug delivery system could enhance its transport to cancer cells (48). The findings of our investigation are in agreement with those of former research that has shown that MET and SIL inhibit the growth and proliferation of the A549 cell line (52–54).

**Figure 7:**
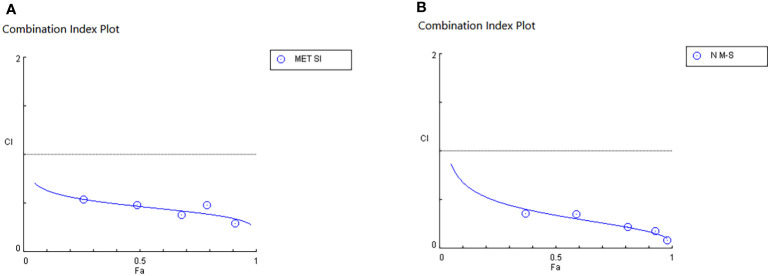
Synergistic inhibitory effects of MET and SIL on the growth of A549 cells. **(A)** F-MET/SIL. **(B)** N-MET/SIL. Combination index (CI) was calculated by isobologram analysis using the Chou–Talalay method. CI = 1, additive effect; CI 1, antagonistic effect. Data represented are from three independent experiments. MET, metformin; SIL, silibinin.

IC50 and combination index (CI50) values for pharmaceutical formulation against A549 cells after 48 h of incubation are shown in **Table 4**, which indicated that N-MET/SIL remarkably have lower IC50 at 48 h. The median-effect approach was used to assess the exact nature of the interaction between MET and SIL in combination form, where CL values greater than, equal to, or less than 1 suggest antagonism, additivity, or synergism effects in the medication combination, respectively (8). The combination of F-MET/SIL and N-MET-SIL had a synergistic influence against A549 cell growth, as shown by the CI50s of F-MET/SIL and N-MET/SIL determined by the combination index chart to be 0.5236 and 0.3409, respectively (**Figure 7**). However, N-MET-SIL has higher synergistic effects against the development of A549 cells.

**Table 4:** IC50 and combination index (CI50) values for the drug formulations against A549 and cells for 48-h incubation time.

Form	Metformin	Silibinin	MET in combination	SIL in combination	CI50
**Pure**	11.95 mM	28.86 μM	7.29 mM	17.36 μM	0.5236
**Nano**	68.42 μM	10.36 μM	27.34 μM	8.14 μM	0.3409

**Figure 8:**
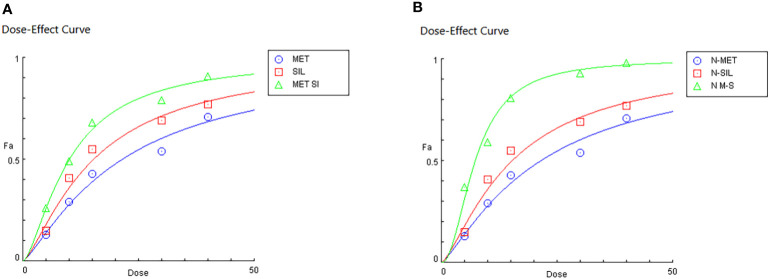
Dose effects of MET and SIL in free form **(A)** and nano-form **(B)**. MET, metformin; SIL, silibinin.

Dose effect evaluation of F-MET, F-SIL, N-MET, N-SIL, and their combination indicated that drugs in combination form have a greater effect than a single form. Moreover, our results showed that the combination of MET and SIL in the niosome has a higher effect on the A549 cell line as shown in **Figure 8**.

### 3.4 Real-time PCR

To examine the anticancer effects of MET and SIL in free and encapsulation forms and their combination in the A549 cell line, the transcription levels of hTERT, BAX, and BCL-2 genes were measured by RT-PCR.

BAX (pro-apoptotic) and BCL-2 (anti-apoptotic) belong to the BCL-2 family, which regulates the apoptosis process through the mitochondrial pathway (55). A recent study has shown that normal expression of BAX in cancer cells was associated with better results, while expression of BCL-2 in tumor cells caused drug resistance. Therefore, it is anticipated that altering the expression of these genes will cause apoptosis and prevent the growth of cancer cells (56).

Abnormal expression of telomerase is found in many cancers that have an essential role in the proliferation and immortality of tumor cells, which is a hallmark of cancer cells. This feature of telomerase becomes a potential target in cancer treatment and drug delivery (57). It is believed that hTERT’s transcriptional modulation, which activates telomerase in a variety of malignancies, is essential. Also, it has been demonstrated that inhibiting hTERT stimulates the activation of apoptosis in cancer cells (47). As a result, discovering a treatment that can both suppress gene expression and trigger apoptosis may be a useful first step in treating lung cancer.

**Figure 9:**
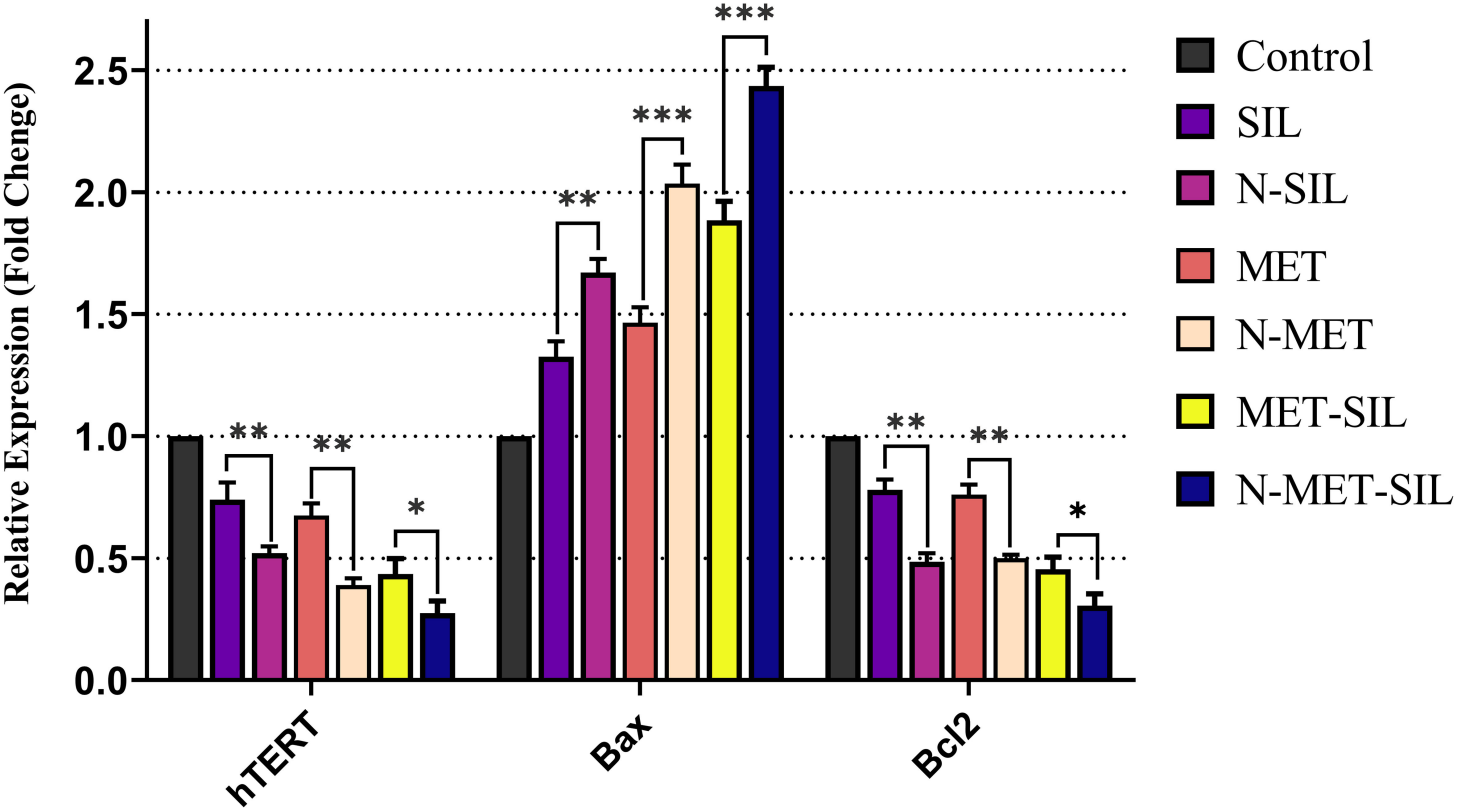
Inhibitory effects of F-MET, F-SIL, their nano-form, and their combination on expression levels of hTERT, BAX, and BCL-2 in A549 lung cancer cells. Data represented are from three independent experiments (***p< 0.001, **p< 0.01, and *p< 0.05). MET, metformin; SIL, silibinin.

In our research, it is found that MET and SIL in free and nano-capsulated forms inhibited the expression of hTERT and BCL-2 while inducing the expression of BAX (**Figure 9**).

Also, our results have discovered that F-MET/SIL and N-MET/SIL downregulate hTERT and BCL-2 and upregulate BAX more than individual forms. This proves that drug combination may enhance anticancer effects and have a synergistic inhibitory impact on tumor cells. This approach could be helpful to increase the therapeutic benefit of individual treatment, particularly in the elimination of obstacles such as tumor resistance and low drug efficiency. Our data are in agreement with those of a recent study that has shown that MET and SIL alone and in combination could inhibit hTERT gene expression (58).

### 3.5 Apoptosis

**Figure 10:**
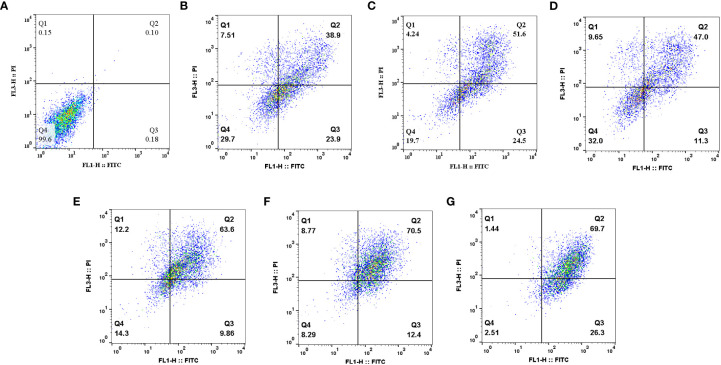
Effects of F-MET, F-SIL, N-MET, N-SIL, and their combination on induction of apoptosis in A549 lung cancer cell line. **(A)** Niosome, **(B)** F-MET, **(C)** N-MET, **(D)** F-SIL, **(E)** N-SIL, **(F)** F-MET-SIL, and **(G)** N-MET-SIL. MET, metformin; SIL, silibinin.

To evaluate the anticancer effect of drugs, an apoptosis assay can be performed (59). Apoptosis is a specifically programmed signaling pathway that induces cell death. Numerous conditions in cancer cells including increased levels of anti-apoptotic proteins and decreased levels of pro-apoptotic proteins cause the apoptotic process to be inhibited, which accelerates cancer cell development and resistance to numerous types of anticancer drugs (60). For this reason, the discovery of treatments that promote the efficient destruction of cancer cells by apoptosis has been one of the clinical oncologists’ purposes for more than three decades (61). Herbal remedies exhibit anticancer efficacy through the stimulation of apoptotic pathways, which is regarded as a novel cancer therapy (60). Recent studies demonstrated that MET and SIL induce apoptosis in most cancer cells (62, 63). In this study, we showed the efficacy of F-MET, F-SIL, N-MET, N-SIL, and their combination on induction of apoptosis in A549 lung cancer cells. Our data indicated that both MET and SIL induce apoptosis in the A549 lung cancer cell line. Results demonstrated that MET and SIL in nano-form induce apoptosis more than an individual one. Also, our findings showed that a combination of F-MET/SIL and N-MET/SIL significantly induces apoptosis and causes cell death in the A549 lung cancer cell line, while N-MET-SIL has a greater effect than F-MET-SIL (**Figure 10**).

In line with our results, Cheng-Chia Tsai et al. have shown that combined treatment of MET and SIL induces apoptosis in colorectal cancer, whereas there was no evidence of apoptosis in colorectal cancer cells when MET and SIL were used alone (7).

### 3.6 Cell cycle

**Figure 11:**
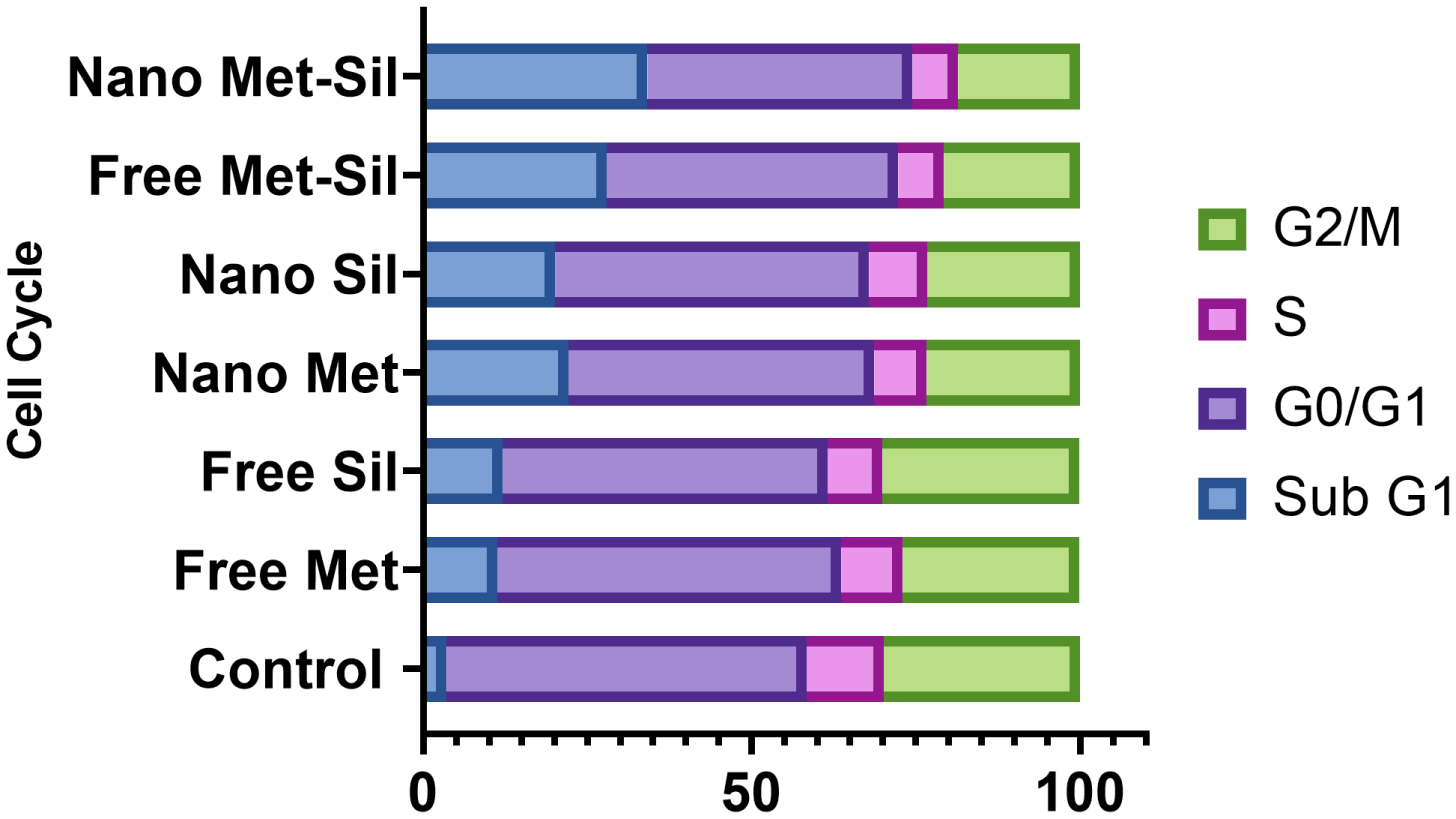
Suppression effects of MET and SIL, their nano-form and their combination on cell cycle of A549 cell line.

**Figure 12:**
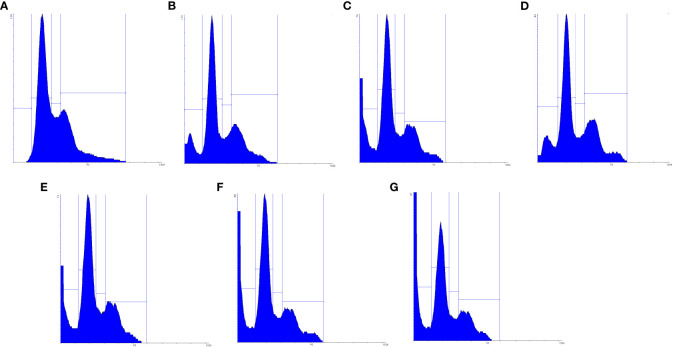
Schematic figure of MET, SIL, F-MET, F-SIL and their combination on cell cycle arrest of A549 cell line. **(A)** Niosome, **(B)** F-MET, **(C)** N-MET, **(D)** F-SIL, **(E)** N-SIL, **(F)** F-MET-SIL, **(G)** N-MET-SIL.

The cell cycle is highly regulated by a group of enzymes, proteins, cytokines, and specific cell cycle signaling pathways, which have a critical role in cellular proliferation and repair processes (64). Continued cell division is a hallmark of cancer cells that is due to mutations that cause damage to cell cycle regulators and cell cycle progression. A necessary consequence is that all cancer cells are related to the non-stop cell cycle. Understanding the mechanisms of the continuous cell cycle in cancer cells could improve therapeutic opportunities to create new combination treatments (65). Recent studies have shown that MET and SIL could induce cell cycle arrest in most cancer cells (62, 66). Dong Hao Jin et al. indicated that MET could promote cell cycle arrest at the G1 phase of the cell cycle (67). Also, Samiha Mateen et al. demonstrated that SIL suppressed the cell cycle at the G1 phase (68). In this research, we assessed the effect of F-MET, F-SIL, N-MET, N-SIL, and their combination and synergistic effect on the cell cycle of the A549 lung cancer cell line. Our data are in agreement with the previous studies mentioned that MET and SIL could suppress the cell cycle at phase G1, and nano-capsulation of these drugs had a higher effect (**Figures 11** and **12**). Also, we demonstrated that the combination of MET and SIL and their nano-form significantly suppress the cell cycle at phase G1.

## 4 Conclusion

In this study, we aimed to synthesize an effective delivery system of PEGylated niosome for metformin and silibinin and evaluated their anticancer effect against A549 lung cancer cells.

The anticancer effect of the metformin and silibinin was increased when they were loaded in PEGylated niosomal NPs compared to the free form. This study exhibited that PEGylated niosomal NPs are an appropriate approach for the effective delivery of drugs for lung cancer cells. Metformin and silibinin in free and encapsulated forms induced apoptosis and cell cycle arrest in cancer cell lines. This evidence suggested that applying a combination of drugs in PEGylated niosomal NPs had a greater effect on inducing apoptosis cell cycle arrest. According to the result of qRT-PCR, the expression of hTERT and BCL-2 genes decreased significantly, while the expression of BAX increased in the treated A549 lung cancer cells. In particular, the downregulation of hTERT and BCL-2 and upregulation of BAX gene expression were enhanced when cells treated with the combination of metformin and silibinin were loaded into PEGylated niosomal NPs. Finally, it can be determined that a combination of metformin and silibinin in PEGylated niosomal NPs is an effective strategy for treating lung cancer, and future studies need to investigate the effects of these drugs in *in vivo* experiments.

## Data availability statement

The data that support the findings of this study are available on request from the corresponding author.

## Author contributions

Writing—original draft preparation: ES-J. Editing: DJ-G and EB. Conceptualization and supervision: NZ. All authors contributed to the article and approved the submitted version.
